# Preparation of Polarity-Marked Microtubules Using a Plus-End Capping DARPin

**DOI:** 10.21769/BioProtoc.5109

**Published:** 2024-11-20

**Authors:** Gil Henkin, Cláudia Brito, Andreas Plückthun, Thomas Surrey

**Affiliations:** 1 Centre for Genomic Regulation (CRG), The Barcelona Institute of Science and Technology, Carrer del Dr. Aiguader 88, Barcelona, Spain; 2 University of Zurich, Department of Biochemistry, Zurich, Switzerland; 3 Universitat Pompeu Fabra (UPF), Barcelona, Spain; 4 Catalan Institution for Research and Advanced Studies (ICREA), Passeig de Lluis Companys 23, Barcelona, Spain

**Keywords:** Microtubules, In vitro reconstitution, Fluorescence microscopy, Microtubule polarity, Microtubule-associated proteins (MAPs), Molecular motors, DARPins

## Abstract

The eukaryotic cytoskeleton is formed in part by microtubules, which are relatively rigid filaments with inherent structural polarity. One consequence of this polarity is that the two ends of a microtubule have different properties with important consequences for their cellular roles. These differences are often challenging to probe within the crowded environment of the cell. Fluorescence microscopy–based in vitro assays with purified proteins and stabilized microtubules have been used to characterize polarity-dependent and end-specific behaviors. These assays require ways to visualize the polarity of the microtubules, which has previously been achieved either by the addition of fluorescently tagged motor proteins with known directionality or by fluorescently polarity marking the microtubules themselves. However, classical polarity-marking protocols require a particular chemically modified tubulin and generate microtubules with chemically different plus and minus segments. These chemical differences in the segments may affect the behavior of interacting proteins of interest in an undesirable manner. We present here a new protocol that uses a previously characterized, reversibly binding microtubule plus-end capping protein, a designed ankyrin repeat protein (DARPin), to efficiently produce polarity-marked microtubules with different fluorescently labeled, but otherwise biochemically identical, plus- and minus-end segments.

Key features

• Produces polarity-marked microtubules with biochemically identical segments

• Allows analysis of end-specific and polarity-dependent activities of purified microtubule-associated proteins

• Requires purified microtubule plus-end capping DARPin (D1)_2_

• Concentrations optimized for porcine brain tubulin

## Graphical overview


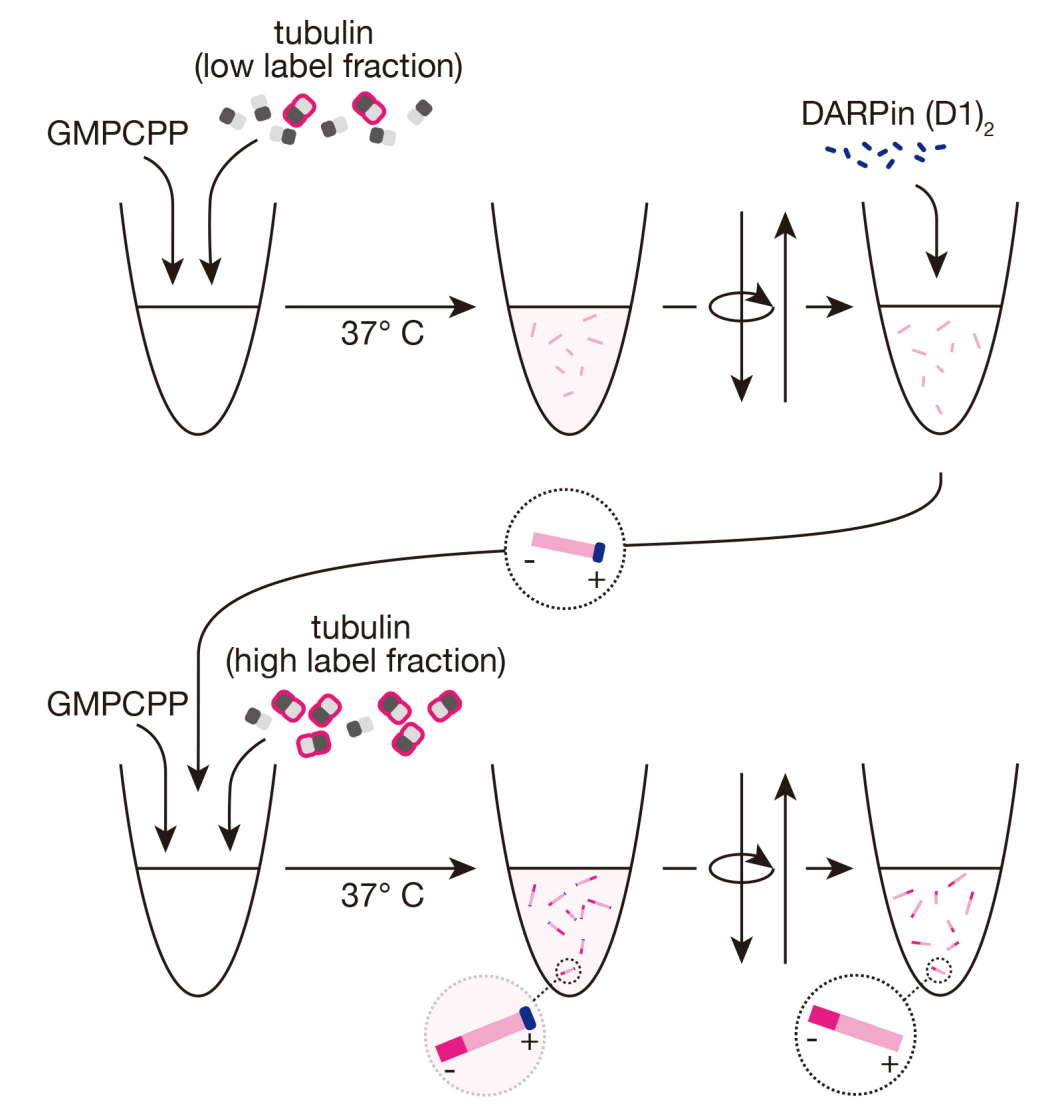


## Background

Microtubule filaments have a structural polarity determined by the orientation of their constituent α/β-heterodimers. When grown in vitro from purified tubulin, microtubules stochastically switch between growing and shrinking at both ends with distinct dynamics [1,2]. Plus-ends, where β-tubulin is exposed, typically grow faster and switch to the shrinking state more frequently than the minus-ends [2,3]. In cells, where a growing list of proteins has been shown to preferentially interact with plus- or minus-microtubule ends [4–6], the minus-end is often, but not always, stabilized by the microtubule nucleating γ-tubulin ring complex [7–9], whereas the plus-end is subject to control by various proteins that control dynamic instability rates [4]. However, the extent to which microtubule-associated proteins selectively interact with one or the other microtubule end is often hard to determine. This is due to the high density of microtubules in structures such as the mitotic spindle or in neuronal axons relative to the resolution of fluorescence microscopy typically used to observe proteins in live cells.

Biochemical assessment in vitro, with purified proteins of interest, can readily reveal end-specific behaviors, amenable to straightforward kinetic and dynamic analyses that are often impossible in cells [4,10–17]. Determining this behavior requires being able to determine unambiguously the polarity of individual microtubules, which is often done through differential fluorescent marking [18–20]. However, the majority of protocols published for selective marking involve use of tubulin chemically modified with *N*-ethylmaleimide (NEM) [18,19,21], which blocks microtubule growth from the minus-end but is also inhibitory to growth at the plus-end, even if less so, requiring careful optimization of modified and unmodified tubulin concentrations and resulting in microtubules that are not fully polymerization-competent in subsequent assays. Furthermore, the protocols involve two steps with two different stabilizing agents, with highly distinct mechanisms of stabilization—the slowly-hydrolyzing GTP analog guanosine-5'-[(α,β)-methyleno]triphosphate (GMPCPP) and the microtubule-binding drug paclitaxel (taxol). This means that the two segments of the microtubule lattice, as well as the ends, are also distinct—one segment contains only GMPCPP-tubulin, while the other contains only taxol-stabilized, partially NEM-modified GDP-tubulin, with differences in chemistry, structure, and mechanical properties [22–27]. These differences can confound the analysis of protein binding to the different microtubule segments and the behavior of potentially end-specific microtubule-binding proteins. Indeed, various microtubule-associated proteins are known to exhibit strikingly different behaviors depending on the nucleotide state of the microtubule lattice [28–31].

We were interested in updating this protocol in such a way that we could produce polarity-marked microtubules where both segments were otherwise biochemically identical. Our procedure is based on recent work that has identified de novo–designed proteins that reversibly cap the growth of distinct microtubule ends [32–34]. We use one of these proteins, a designed ankyrin repeat protein (DARPin) in a tandem arrangement, termed (D1)_2_, to provide a new method for polarity marking stable microtubules that are grown with GMPCPP-only throughout. Furthermore, we show how to confirm the polarity of the microtubules, using a simple gliding assay with a microtubule motor protein of known directionality.

## Materials and reagents

**Reagents**

1. Porcine brain tubulin, prepared as previously reported [35,36]
2. Purified tandem DARPin D1 protein [(D1)_2_], expressed in *E. coli* and purified according to standard DARPin expression and purification protocols [37,38]. A detailed description of the (D1)_2_ expression on a 500 mL scale and purification via metal affinity chromatography and size exclusion chromatography can be found here [14,32]. The bacterial (D1)_2_ expression plasmid can be requested from A. Pluckthun (plueckthun@bioc.uzh.ch)
3. Fluorescently labeled porcine brain tubulin (i.e., AlexaFluor647 or Atto647-labeled) prepared as previously reported [21,35]
*Note: Fluorescence labeling ratio should be determined by Nanodrop prior to starting the protocol.*
4. Optional for neutravidin-biotin-based immobilization in, e.g., TIRF microscopy assays: biotinylated porcine brain tubulin, prepared as previously reported [21,35]
5. Piperazine-*N,N*′-bis(2-ethanesulfonic acid) (PIPES) (Sigma-Aldrich, catalog number: P6757)
6. Magnesium chloride (MgCl_2_), 1 M (Sigma-Aldrich, catalog number: 63069)
7. Ethylene glycol-bis(β-aminoethyl ether)-*N,N,N′,N*′-tetraacetic acid (EGTA) (Sigma-Aldrich, catalog number: E3889)
8. Guanosine-5'-[(α,β)-methyleno]triphosphate (GMPCPP), 10 mM (Jena bioscience, catalog number: NU-405)
9. High-quality purified water, e.g., Millipore Milli-Q

*For gliding assay:*

1. An active, directional microtubule motor protein; we had the minus-end-directed HSET motor protein available in the laboratory, prepared as previously published [39]. Many other microtubule motors with established polarity, such as the plus-end directed kinesin-1 or its motor-domain truncations, can be used instead
2. Poly(L-lysine)-polyethylene glycol (PLL-PEG) [SuSoS, catalog number: PLL(20)-g[3.5]- PEG(2)]
3. β-Mercaptoethanol (β-ME), 14.3 M (Sigma-Aldrich, catalog number: M6250)
4. β-casein (Sigma-Aldrich, catalog number: C6905)
5. ATP (Sigma-Aldrich, catalog number: A6419)

**Solutions**

1. EGTA, 0.5 M, pH 8 (see Recipes)
2. BRB80, 5× stock (see Recipes)
3. BRB80, 1× (see Recipes)

*For gliding assay*:

1. PLL-PEG, 2 mg/mL (see Recipes)
2. β-casein, 25 mg/mL (see Recipes)
3. ATP, 100 mM, pH 7 (see Recipes)
4. Gliding buffer (see Recipes)

**Recipes**

*Note: All solutions should be prepared using high-quality purified water, e.g., Millipore Milli-Q.*

1. **EGTA, 0.5 M, pH 8**
  Add solid KOH to dissolve EGTA powder and adjust pH only by adding KOH, being careful not to overshoot; store at room temperature.
2. **BRB80, 5**× **(50 mL)**
  **Table:** 

Reagent	Final concentration	Quantity or Volume
PIPES	400 mM	6.06 g
EGTA (0.5 M, pH 8.0)	5 mM	0.5 mL
MgCl_2 _(1 M)	5 mM	0.25 mL
Total		50 mL, pH 6.8
  Filter (0.22 μm pore size) and store for up to 4 weeks at 4 °C.
*Note: PIPES dissolves upon the addition of solid KOH. MgCl_2_ and EGTA should be added only after PIPES is dissolved (requiring ~1.35 g of KOH for these amounts). The final pH should be adjusted only by addition of KOH, being careful not to overshoot, to control the total ionic strength of the solution.*
3. **BRB80, 1×**
  Diluted from the above recipe on the day of use; keep on ice.
4. **PLL-PEG, 2 mg/mL, in water**
  Aliquot and store at -20 °C.
5. **β-casein, 25 mg/mL, in BRB80**
  Ultracentrifuge, aliquot, snap-freeze in liquid nitrogen, and store at -80 °C.
6. **ATP, 100 mM, pH 7**
  Filter, aliquot, and store at -80 °C.
7. **Gliding buffer**
  BRB80 with 5 mM β-ME, 1 mM ATP, 1 mg/mL β-casein; prepare on the day of the experiments and keep at room temperature.

**Laboratory supplies**

1. 1.5 mL centrifuge tubes (e.g., Eppendorf, catalog number: 0030120086)
2. Ice bucket
3. Pipettes
4. Pipette tips

*For imaging/ gliding assay:*

1. Cover glasses, thickness #1.5, 22 × 22 mm (VWR, catalog number: 630-1843)
2. Standard microscopy slides, 76 × 26 mm (e.g., VWR, catalog number: 631-1550P)
3. Double-sided adhesive fleece transparent tape, 10 m × 15 mm (Tesa^®^, catalog number: 053380000001)
4. Filter paper 3 mm CHR, 46 cm × 57 cm (Whatman^®^, catalog number: 3030-917)
5. Korasilon^TM^ Paste, medium viscosity (Sigma-Aldrich, catalog number: 769681-1EA)
6. Diamond scribing pen (e.g., Ted Pella, catalog number: 54468)
7. Forceps (e.g., Sigma-Aldrich, catalog number: Z680214-1EA)

## Equipment

1. Microcentrifuge (e.g., Eppendorf, model: 5418 R)
2. Heat block set to 37 °C (e.g., Eppendorf, model: ThermoStat C)

*For imaging/ gliding assay:*

1. Fluorescence microscope (e.g., Nikon Instruments, model: E600)

## Software and datasets

1. ImageJ/FIJI (e.g., ImageJ version 1.54, https://imagej.net/ij/index.html)

## Procedure

- **Dim, GMPCPP-stabilized microtubules**
  1. Thaw aliquots of frozen unlabeled tubulin, fluorescent tubulin, and (optionally) biotinylated tubulin. On ice, prepare a mixture of 3 μM total tubulin in BRB80 1×, supplemented with 0.5 mM GMPCPP, including fluorescent tubulin at a final labeling ratio of 3%–5% and (optionally) 18% biotinylated tubulin, with a total volume of 200 μL in a microcentrifuge tube.
  2. Incubate mixture on ice for 5 min.
  3. Transfer the tube to 37 °C and incubate for 1 h. Warm 3 mL of BRB80 1× to 37 °C for subsequent steps.
  4. After incubation, dilute seeds with 170 μL of warm BRB80 1× and centrifuge at 17,000× *g* for 10 min at room temperature in the tabletop microcentrifuge.
  5. Remove the supernatant.
*Note: You should be able to see a pellet with color depending on the fluorescent dye used.*
  6. Wash the pellet by adding 400 μL of warm BRB80 1× and centrifuging again at 17,000× *g* for 10 min at room temperature.
  7. Remove the supernatant and resuspend the pellet in 50 μL of warm BRB80 1×. Keep microtubules at room temperature.
- **Bright GMPCPP-stabilized minus-end extensions using DARPin (D1)_2_**
  1. Thaw an aliquot of DARPin (D1)_2_ and dilute to 2.5 μM in BRB80 1× on ice.
  2. From freshly thawed aliquots of unlabeled tubulin, fluorescent tubulin, and (optionally) biotinylated tubulin, prepare a new “bright” mixture of 3 μM total tubulin in BRB80 1×, supplemented with 0.5 mM GMPCPP, including fluorescent tubulin at a final labeling ratio of 10%–15% and (optionally) 18% biotinylated tubulin, with a total volume of 200 μL in a microcentrifuge tube.
  3. Incubate the “bright” mixture on ice for 5 min.
  4. During the incubation, add 2 μL of the DARPin (D1)_2_ dilution to 8 μL of the previously prepared dimly-labeled microtubules at room temperature. The protein will bind the microtubule plus-ends, ensuring that the subsequent bright extensions are exclusively from the microtubule minus-ends.
  5. Dilute the “bright” mixture with 200 μL of cold BRB80 1×.
  6. Transfer 90 μL of the diluted “bright” mixture to 37 °C and incubate for 1 min.
  7. Add the 10 μL DARPin (D1)_2_/dim seed mixture directly to the warmed “bright” mixture [resulting in final concentrations of 50 nM DARPin (D1)_2_, 1.35 μM tubulin, and 0.23 mM GMPCPP].
  8. Incubate the mixture at 37 °C for 1 h. Brightly labeled, stabilized extensions will grow from microtubule minus-ends, as the plus-ends are capped, resulting in microtubules with two distinctly labeled segments.
  9. After incubation, dilute seeds with 170 μL of warm BRB80 1× and centrifuge at 17,000× *g* for 10 min at room temperature in the tabletop microcentrifuge.
  10. Remove the supernatant, including unpolymerized tubulin, GMPCPP, and the capping protein, DARPin (D1)_2_.
*Note: There are fewer seeds at this point, so the pellet may be challenging to see.*
  11. Wash the pellet by adding 400 μL of warm BRB80 1× and centrifuging again at 17,000× *g* for 10 min at room temperature.
  12. Remove the supernatant and resuspend the pellet in 50 μL of warm BRB80 1×. Keep microtubules at room temperature. Polarity-marked microtubules should be used within the day.
- **Quick check: properly segmented microtubules**
  1. Using a fluorescent microscope, the success of the protocol can be checked by quickly preparing a 1:6 μL dilution of the microtubules and “squashing” the dilution between a coverslip and slide. Fluorescence imaging will readily show how many of the dim stabilized microtubules have a single bright extension. (Figure 1).
    **Figure 1.**
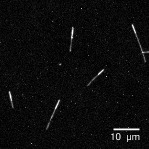
AlexaFluor647-labeled, polarity-marked microtubules on a glass slide. Imaged with a 60*×* objective on a spinning disk confocal fluorescence microscope.
- **Gliding assay to determine correct polarity labeling**
  We use a simple gliding assay adapted from previous work to determine the polarity of our labeled microtubules.
  1. Place two strips of the double-sided tape on a standard microscopy slide (the counter glass) about 5 mm apart. Press the tape down forcefully with forceps or the end of a microcentrifuge tube. Leave the protective backing.
  2. Leave for at least 15 min at room temperature to allow the tape to fully adhere.
  3. Pipette 10 μL of 2 mg/mL PLL-PEG between the two strips of tape and spread to all the edges using a pipette tip.
  4. Allow PLL-PEG solution to dry.
  5. Rinse thoroughly with MilliQ water and dry using pressurized air.
  6. Using a diamond scribe pen, cut a #1.5 22 × 22 mm coverslip into four equal squares. Any debris from the cutting can be removed with pressurized air.
  7. Remove the protective tape backing and place one of the coverslip pieces on top, pressing down firmly over the tape to ensure proper adhesion.
  8. Use 50 μL of gliding buffer to wash the chamber, using filter paper to pull the solution through, and incubate with gliding buffer for 3 min at room temperature.
  9. Dilute the motor protein, e.g., the minus-end-directed kinesin-14 HSET, to 100 nM in gliding buffer, and wash 50 μL through the chamber, followed by 3 min incubation with the motor dilution at room temperature.
  10. Dilute the polarity-marked seeds 1:6 with room temperature gliding buffer and flow 50 μL into the chamber, again pulling through with the filter paper.
  11. Seal the chamber ends with Koralison^TM^ paste.
  12. Image fluorescent microtubules at the microscope, using a 5 s interval between frames to capture gliding events over the course of 20 min (Figure 2, Video. 1). Seeds should glide directionally according to the motor, e.g., the minus-end-directed HSET will drive microtubules with the plus/dim end leading. We found that 100% of motile microtubules with one dim and one bright segment glided in the appropriate direction. It can be difficult to achieve such a high percentage of correctly labeled polarity-marked microtubules using the NEM-tubulin method, which typically requires defining an acceptable threshold for successful labeling that is sufficiently high for the specific downstream assay.
    **Figure 2.**
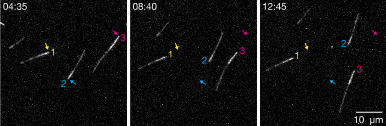
AlexaFluor647-labeled, polarity-marked microtubules gliding on glass-adsorbed HSET motor proteins. As a minus-end-directed motor, HSET will drive microtubules according to their polarity with their plus-ends leading, indicating that the bright and dim segments are the minus- and plus-ends of the microtubule, respectively. Imaged with a 60*×* objective on a spinning disk confocal microscope. Arrows indicate the position of the bright end in the first frame. See also Video 1.
    **Video 1.**
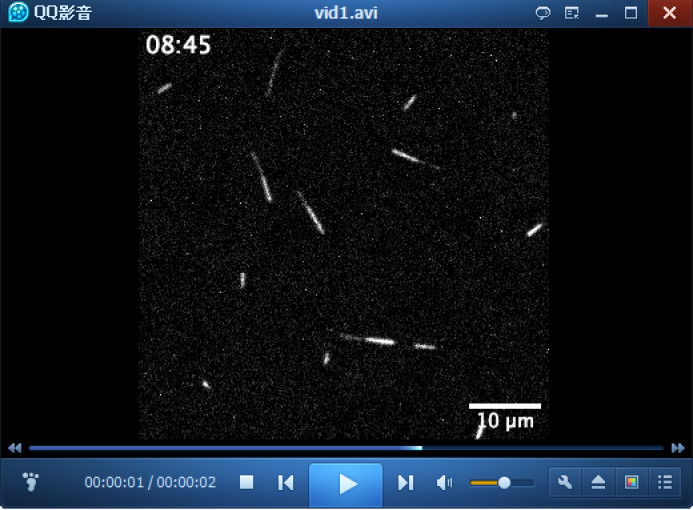
Polarity-marked microtubules gliding on glass-adsorbed minus-end-directed HSET motor proteins. Microtubules attach to the surface over time and glide in the direction of their dim segments, i.e., with their plus-ends leading. Imaged with a 60× objective on a spinning disk confocal fluorescence microscope.

## Data analysis

Data can be analyzed relatively simply by inspection of the fluorescent images and videos. Counting the number of appropriately segmented microtubules (one bright and one dim segment) vs. the overall number of microtubules gives a measure of efficiency. Proper gliding can be inferred by the directionality of the motor protein. In the case of surface-bound kinesin-14, a minus-end-directed motor, microtubules will be driven to glide by their plus-ends first, which should be the dim end according to this protocol.

## Validation of protocol

This protocol has been used and validated in the following research article:

- Henkin, Brito et al. [14]. The minus-end depolymerase KIF2A drives flux-like treadmilling of γTuRC-uncapped microtubules. J Cell Biol (Figure 1, panels C, D, F-K; Figure S2, panels B, C)

## General notes and troubleshooting

**General notes**

1. In our hands, using the gliding assay, we found that 100% of microtubules with appropriate segmentation (one dim section, one bright section) were indeed correctly polarity labeled: n = 37 microtubules across two experiments, as we previously published [14]. As further validation, we showed that KIF2A, a microtubule depolymerase with a preference for minus-ends (independently confirmed by other groups [13,40]), depolymerized 98% of polarity-labeled microtubules predominantly from the bright minus-end (n = 271 microtubules across three different experimental sets) [14].
2. These seeds, if biotinylated, can be used in assays with microtubule-associated proteins, as in our previously published manuscript [14].
3. More details on the procedure for preparing these experiments using biotinylated, passivated glass and neutravidin links can be found in previous work [35].

**Troubleshooting**

Problem 1: Microtubule extensions do not grow during the DARPin (D1)_2_ step.

Possible cause: Tubulin concentration is too low; dynamic properties of tubulin may vary according to source, preparation methods, or means of storage.

Solution: Test in parallel a range of tubulin concentrations to grow the bright extensions. We tested a range between 0.2 and 1.5 μM to find a good condition that promotes relatively fast growth without nucleating too many new microtubules.

Possible cause: DARPin (D1)_2_ concentration too high; the protein acts by capping exposed β-tubulin, not only at the microtubule plus-end but also in solution, resulting in suppression of minus-end growth as well at high concentrations.

Solution: Test in parallel a range of DARPin (D1)_2_ concentrations. We tested a range from 50 to 150 nM to identify a condition that blocks plus-end but allows minus-end growth.

Problem 2: Microtubules extend from both directions during the DARPin (D1)_2_ step.

Possible cause: Tubulin concentration is too high.

Solution: Test in parallel a range of tubulin concentrations (see above).

Possible cause: DARPin (D1)_2_ concentration too low.

Solution: Test in parallel a range of DARPin (D1)_2_ concentrations (see above).

Problem 3: Microtubules show a repeated dim-bright-dim-bright patterning.

Possible cause: Microtubules can anneal end-to-end during storage at room temperature.

Solutions: Dilute to slow further annealing. Resuspend in the final step in 100 or 200 μL of warm BRB80 1× instead of 50 μL. Ensure that microtubules are prepared directly before experiments.

Problem 4: Microtubules do not glide in the gliding assay.

Possible cause: Motor protein not active in given concentrations.

Solutions: Try a different motor protein; try higher concentrations of motor protein; try lower concentrations of motor protein (in case of jamming).
